# Interpretable machine learning approach for neuron-centric analysis of human cortical cytoarchitecture

**DOI:** 10.1038/s41598-023-32154-x

**Published:** 2023-04-05

**Authors:** Andrija Štajduhar, Tomislav Lipić, Sven Lončarić, Miloš Judaš, Goran Sedmak

**Affiliations:** 1 School of Public Health “Andrija Štampar”, School of Medicine, University of Zagreb, 10000 Zagreb, Croatia; 2 Croatian Institute for Brain Research, School of Medicine, University of Zagreb, 10000 Zagreb, Croatia; 3 Laboratory for Machine Learning and Knowledge Representation, Ruder Bošković Institute, 10000 Zagreb, Croatia; 4 Faculty of Electrical Engineering and Computing, University of Zagreb, 10000 Zagreb, Croatia

**Keywords:** Computational neuroscience, Image processing, Machine learning, Brain

## Abstract

The complexity of the cerebral cortex underlies its function and distinguishes us as humans. Here, we present a principled veridical data science methodology for quantitative histology that shifts focus from image-level investigations towards neuron-level representations of cortical regions, with the neurons in the image as a subject of study, rather than pixel-wise image content. Our methodology relies on the automatic segmentation of neurons across whole histological sections and an extensive set of engineered features, which reflect the neuronal phenotype of individual neurons and the properties of neurons’ neighborhoods. The neuron-level representations are used in an interpretable machine learning pipeline for mapping the phenotype to cortical layers. To validate our approach, we created a unique dataset of cortical layers manually annotated by three experts in neuroanatomy and histology. The presented methodology offers high interpretability of the results, providing a deeper understanding of human cortex organization, which may help formulate new scientific hypotheses, as well as to cope with systematic uncertainty in data and model predictions.

## Introduction

The human cerebral cortex is a highly organized, complex structure composed of billions of neurons. One of the most prominent features of the human cerebral cortex are cortical layers—laminar structures parallel to the surface of the cerebral hemisphere and superimposed one on top of the other. This layered structure is caused by variations in cell density, size, and shape of neurons, specific for each cortical layer. The entire cerebral cortex can be subdivided, based on the number of layers, into a six-layered neocortex (or isocortex) and allocortex which can further be subdivided into a two-layered paleocortex, three-layered archicortex and usually a five-layered mesocortex. Today, the most used classification of the neocortical layers is one based on the concept developed by Korbinian Brodmann at the beginning of the 20th century^1^. In this classification, the neocortex is composed of six layers differentiated by neuronal features such as neuronal type, number, size, shape, density, etc. In his seminal work, Brodmann also summarized previous work done on the composition of the neocortex showing that in the generic neocortex, researchers differed significantly in describing the number of layers ranging from four to seven. Thus, we can infer that cortical layers, although biological features of the cerebral cortex, are delineated by arbitrary criteria developed by human observers. Furthermore, the composition, size, and number of layers are not constant throughout the cerebral cortex. Based on variations of these cytoarchitectonic features cerebral cortex can be divided into smaller cortical, cytoarchitectonic areas. At the beginning of the 20th-century researchers in the field of cytoarchitectonics (i.e. study of the cortical building plan) developed several cytoarchitectonic maps which divided the cerebral cortex into smaller structural units with two of the most influential being one developed by Brodmann^1,2^ and the other by von Economo and Koskinas^3^. For every cytoarchitectonic area, a clear set of features can be defined which distinguishes them from other areas. However, borders between two areas are not always clear cut, but are, rather, an area of transition with gradual changes from one to the other. In these transitioning parts it is often difficult for the human observer to precisely and consistently delineate both cortical areas and laminas within areas. Interest in the analysis of these structures is driven by the evidence of the relationship between features of cytoarchitectonic structure and cortical functions. Today, it is believed that the way neurons are distributed in the brain determines its function. The subtleties in this fine structure of the brain underlying its function can be characterized in great detail by studying the organization of cells across the cortex^4^. However, investigations in this field are mostly done manually, require a significant amount of researchers’ time, introduce observer-dependent bias and hinder the reproducibility of the research^5^. As technology advances, more and more digitized histological data becomes available. Computer-aided methods provide means for faster, more objective, and higher-throughput investigations of the cortical structures through automatized processing of histological sections of the cortex. This enables researchers to answer various scientific questions by better understanding the anatomical and functional organization of the brain, as well as observing subtle changes in the brain structures caused by neurological and psychiatric diseases.

Ever since the first methods which introduced automation in the analysis of cortical layers, the central idea was the sampling of different tissue measures along transverse lines drawn either manually or semi-automatically across the cortex, perpendicular to the laminar structure and spanning the full width of the cortex^6–9^. An important step was the development of gray-level index (GLI)^10^, a method that measures areal fractions of darkly stained cell bodies across the cortex, yielding different neuron density profiles depending on the location of interest and identifies positions at the cortical ribbon where cytoarchitectonic features change^11^. Blocks of adjacent profiles may be represented by feature vectors and compared between areas of the cortex. Profile features were used for the estimation of cell counts between cortical areas, providing realistic information about neuron density^12^. Higher levels of automation enabled faster analysis of larger datasets. The BigBrain is a high-resolution 3D digital atlas of the whole human brain, providing huge amounts of high-resolution histological data for neuroanatomical studies^13^. The GLI profiles were combined with machine learning methods to create laminar segmentation on the BigBrain dataset, creating parcellations across large brain areas^14,15^. Cortical layers were also segmented using convolutional neural networks^16^, and a comparison with GLI approach is given^17^. The first article that uses features and statistics of individual neurons appeared in 2017, where the authors used automatic segmentation of cells in the mouse brain and analyze cellular shape statistics without machine learning models^18^. In the year 2018, the first approach that does not use profiles across the cortex was proposed^19^. A combined approach of unsupervised and supervised machine learning was used on a dataset of 2-photon microscopic images of the rat cortex. One can observe the transition towards automation, analysis of larger datasets, and usage of machine learning methods in the field that was until recently dominated by the usage of classical image processing techniques that use various filtering, image-wide pixel transformations, thresholding, and similar operations.

Machine learning-based methods rely on a training dataset to develop their predictive capabilities, allowing them to generalize and make predictions on unseen data. In this context, the unseen data refers to portions of tissue that have not been manually delineated or labeled by human researchers. In machine learning, such an approach is known as supervised learning, sometimes also referred to as predictive modeling. Having adequate learning data is essential in developing successful models, and human labels are considered the gold standard. However, over the years, the impact of human bias in brain parcellation has been increasingly recognized and many methods sought to overcome this issue by developing objective quantitative measures and usage of statistics to distinguish between different layers and brain areas^20^. In a recent paper^21^, authors use neuronal density estimates to infer local neuronal connectivity while addressing the issue of human bias in the manual segmentation of cortical layers and use an unsupervised clustering approach to identify and represent the laminar structure. An important aspect to consider in the analysis of images of the human brain, or biomedical images in general, is to what extent automated systems should recreate the work of human investigators. Computer vision systems can give access to underlying image contents that are not visible, process every image equally, and provide partial or complete automation of the process^22^, especially concerning the recently available massive amounts of high-resolution and multimodal data, far beyond the capabilities of any kind of manual analysis. Such systems can derive and analyze detailed anatomical and biologically meaningful information on a large scale and reveal currently neglected structuring principles and provide a deeper understanding of the laminar structure. This suggests that there is a need to move beyond the limitations of manually created parcellation in conventional atlases towards data-driven analysis. Ideally, a representation of a histological section that would contain information to enable objective and unsupervised classification or parcellation of layers and even reveal sub-layering would help resolve many unanswered issues in the study of brain anatomy and physiology.

In this paper, we hypothesize that the current methods do not offer such capabilities due to their inability to capture subtle cytoarchitectonic features. Consequently, they express low explainability and interpretability of their results. Methods based on deep that operate on striding windows across the image may provide convincing parcellations, but we cannot ask what tissue characteristics lead to these results. Here, we investigate the possibility of developing neuron phenotyping^23^ that captures cytoarchitectonic details and can be used for inference about the brain structure using only local tissue information at the cellular level. The usability of this approach is demonstrated through the task of distinguishing cortical layers by classifying each neuron within the six cortical layers and white matter using a supervised machine learning method. The method uses phenotype characterization as an input and predicts the individual neuron’s layer, natively offering a high level of interpretability. We also demonstrate the human ability to distinguish between the cortical layers and explore how learning-based methods can generalize from such noisy labels. The developed framework provides a capability to investigate which neuronal features are characteristic for different areas and presents a prospect for future investigations in the field of cytoarchitectonics.

## Materials and methods

**Figure 1:**
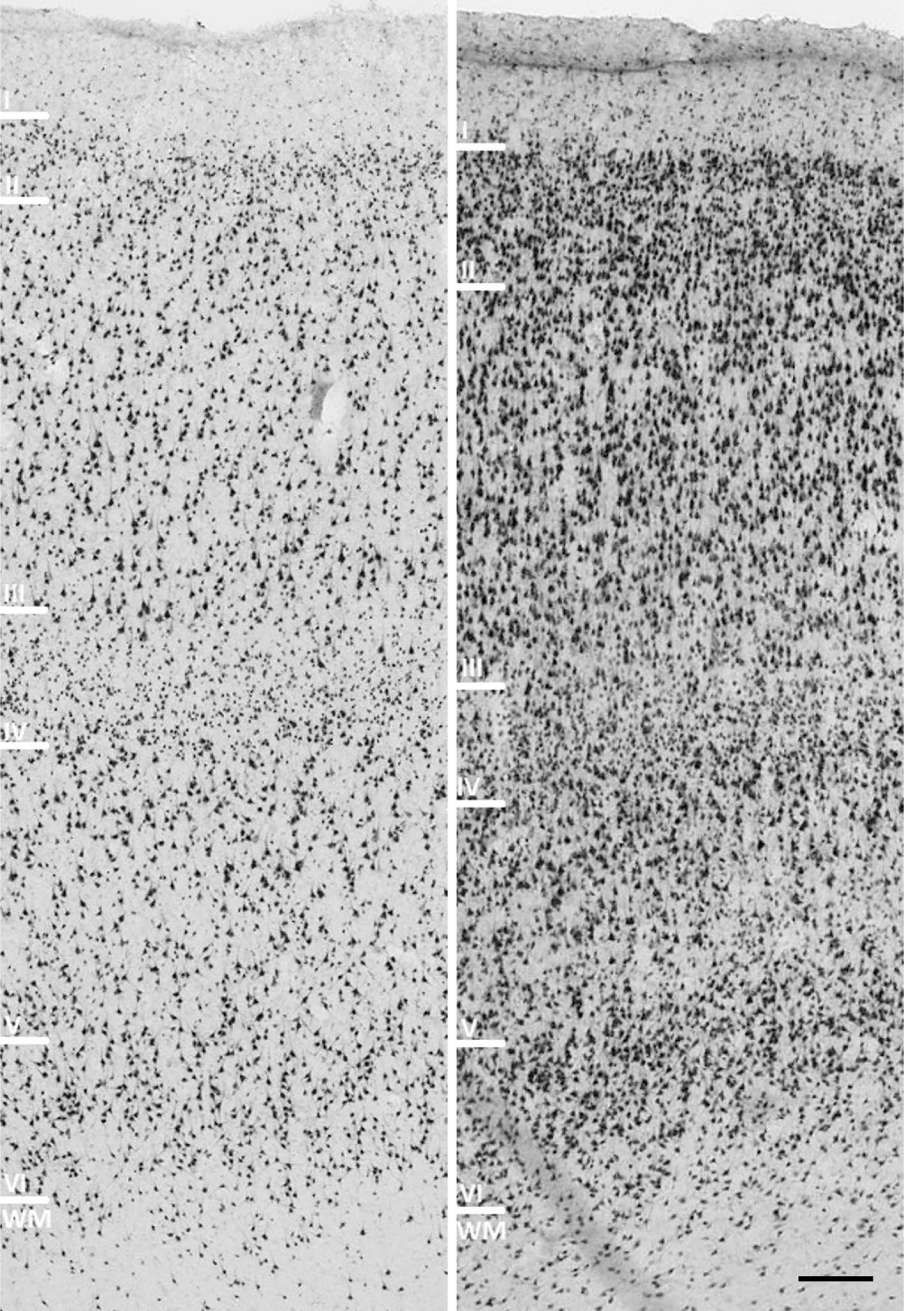
Histological slices stained with NeuN immunohistochemistry method showing varying neuronal morphology and cellular distribution across the layers of the cortex. The left image is taken from the dorsolateral part of the prefrontal cortex (thickness $10\;\upmu \hbox {m}$) and the right image was taken from the orbital part of the prefrontal cortex (thickness $20\;\upmu \hbox {m}$). Scale bar $100\;\upmu \hbox {m}$.

Histological data were obtained from the Zagreb Neuroembryological Collection^24^. Samples used in this study were taken from the prefrontal cortex of two brains (brain 1 55 years old female, post-mortem delay 24h; brain 2 age not available; male, post-mortem delay 4h). Sections were taken from the dorsal and ventral part of the typical six-layered homotypic isocortex of the prefrontal cortex^3,4^. Brains were fixed in $4\%$ PFA for two weeks. Following sampling, sections were dehydrated through a series of ethanol and embedded in paraffin. Sections were cut using rotating microtome at thicknesses of $10\;\upmu \hbox {m}$ and $20\;\upmu \hbox {m}$. The tissue was stained using the NeuN immunohistochemistry method according to standard protocol^25^. NeuN is an RNA-binding nuclear protein, derived from the RBFOX3 gene, which regulates alternative splicing in neurons and is expressed explicitly in all neurons of used tissue specimens. In the experiments, $10\;\upmu \hbox {m}$ and $20\;\upmu \hbox {m}$ sections were used in order to test whether tissue thickness would impact the results. Histological sections were digitized using the Hamamatsu Nanozoomer 2.0 scanner (Hamamatsu Photonics, Japan) at 40x magnification, corresponding to $0.226\mu \hbox {m/pixel}$ resolution. Example histological sections in Fig. 1 show varying neuronal morphology and cellular distribution across the cortical layers. Computational experiments were performed using custom scripts written in Python 3.8 and standard publicly available libraries.

### Neuron-level features

**Figure 2:**
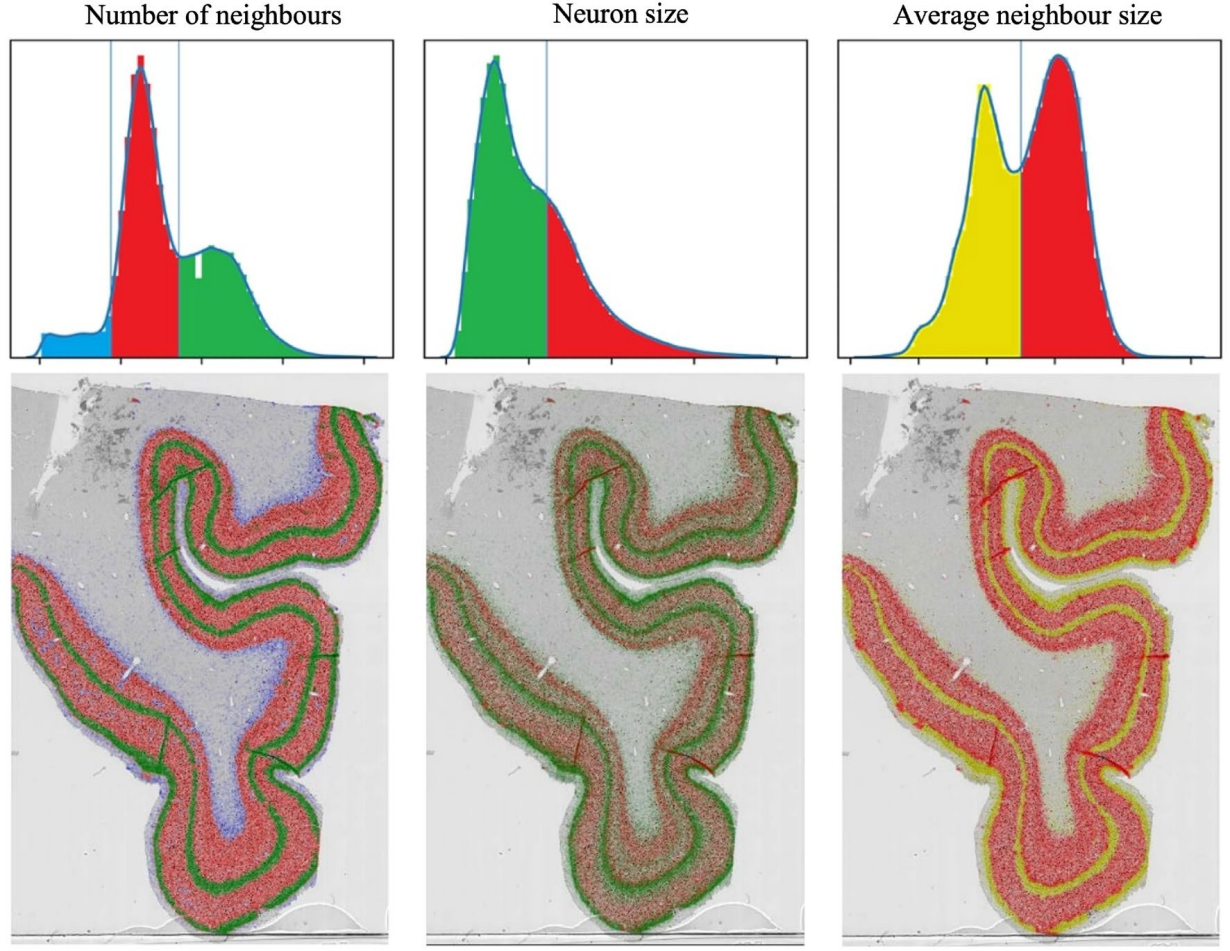
By visualizing the basic neuronal characteristic, the area, and local density, the laminar structure is revealed, as these features exhibit multimodal distributions. Left: Three types of neurons were distinguished by cell density in their surroundings: very sparse (blue), sparse (red), and dense (green). Middle: Larger (red) and smaller (green) neurons. Right: Average size of neighboring neurons is a feature obtained from the previous two: nearest neighboring neurons and their areas, which can also be used to facilitate layer prediction. Here, thresholds that separate the distributions were obtained using minimization of intraclass variance^26^.

In manual delineation, the density and size of neurons are the most important characterizations of the laminar structure. Based on anatomical descriptions and kernel density estimates, three populations of similar densities are assumed (layers II and IV as dense, layers III, V, and VI as average, and Layer I and white matter as sparse), two populations of similar sizes (layers III, V and VI containing on average larger neurons, and layers I, II, IV and white matter containing on average smaller neurons). By plotting a histogram of neuron densities and sizes, one can observe that the features express a multimodal distribution, which can be separated using the minimization of intraclass variance^26^. Figure 2 shows a visualization of separating the neuron populations across the histological section, revealing the laminar structure. In contrast to the classical pixel-based approach to cortical layer segmentation, we used neuron-level tissue descriptors to characterize and examine the underlying tissue properties. We develop several feature classes that describe each neuron in the tissue and use a machine learning model to determine the layer of individual neurons. By classifying all neurons in the tissue, we obtain the parcellation of the laminar structure. To the best knowledge of the authors, this is the first *bottom-up* approach in the analysis of brain cytoarchitectonics that builds from the cellular level and infers about larger structures based on morphological and textural features of individual neurons. Here, we discuss the development of those features, as well as some rationale behind the choices made in their development and selection.

The first step in obtaining the neuron characterization is the segmentation of neurons from the background tissue. The segmentations were obtained using automated methods^27,28^ which use grayscale-guided watershed on anisotropically diffused images to separate neurons, rather than often used distance maps obtained from the grey-level threshold, providing a binary image of segmented, non-overlapping neuron areas. As the goal here is to create consistent results across the tissue, other segmentation methods may be used as well, such as a recently proposed instance segmentation via contour proposals^29^, especially for different staining methods. This step yielded the locations and segmentations of neurons, from which other neuronal characteristics are developed.

Secondly, neuron segmentations were analyzed using ImageJ particle analysis pipeline^30^. An overlap of binary segmentations and the original image was made, and ImageJ’s function *analyze particles* was used to produce measurements of neurons’ bodies. Those were the area, perimeter, circularity, roundness, and Feret’s diameter as well as the mean, median, skewness, and kurtosis of the gray values. More details on particle measurement can be found in ImageJ’s documentation^31^. These features form the basis for investigations in brain microanatomy, as they are often, although not at this level of precision, perceived by the eye of neuroanatomists. By this, we obtain the first neuronal characteristics, which may be visualized to reveal patterns of their appearance across the cortical layers, as shown in Fig. 2. It should be noted that values based on image intensity were not used in the further analysis as it was concluded that these were not usable generally, as they may be heavily influenced by uneven staining across the section and exhibit different values for different staining procedures. These simple measurements do not possess a discriminative power to create clear classifications of neurons within the layers. Therefore, richer descriptors that incorporate neuron neighborhoods were computed, as described below.

It is worth mentioning that density-based clustering algorithms are often used to segment the areas of similar point densities^32–34^, which may be brought into relation with cortical layers having a roughly uniform density within each layer. However, it seems that the neuron distribution in the cortex is such that their intrinsic structure may not be clustered by a single set of global density parameters, as used in clustering methods. Nevertheless, these methods provided insight into some cortical properties. Meaningful clusters were created when considering neurons within the radius between $100\;\upmu \hbox {m}$ and $300\;\upmu \hbox {m}$, containing between 300 and 800 neurons. This lead to the conclusion that the changing nature of neuron distribution in the brain is best characterized when performing measurements in this range. This range approximately also corresponds to the biological limits of interlayer distances. It is important to emphasize that although choosing a predefined radius or a number of neighbors may seem equivalent, analysis of nearest neighbors is preferred over the fixed radius approach. A predefined radius may be interpreted differently, depending on the image resolution. The specified range around a neuron allows for a more detailed and precise analysis of the microstructure of the tissue in that specific area by balancing between obtaining enough information to capture local tissue properties and not being confounded by reaching too far from the neuron toward other layers and incorporating information which is not in the neuron’s vicinity and is, therefore, less relevant for neuron’s phenotype. Also, if a fixed maximum number of neighbors for each neuron is used, efficient data structures like kd-trees^35,36^ may be precomputed. Considering the large number of neurons found in a histological section, efficiency may be of critical importance.

To measure properties of neurons’ neighborhoods, nearest *k* neighbors were considered, for $k \in [50,100,250,500,1000]$. The distances to a neuron’s *k*-th nearest neighbor were used as a feature, as well as their mean, max, min, skewness, kurtosis, and entropy. Basic measures of individual neurons were computed in a similar fashion to produce, for instance, the average area of neighboring 100 neurons, as shown at the right in Fig. 2. A convex hull of neurons’ *k*-neighbours gives information about the area around a neuron and a number of its neighbors and is described using hull area, perimeter, average nearest distance for neurons found in the hull, and standard deviation of nearest distances. Dispersion of neurons may be quantified using *nearest neighbor index* (NNI), a measure that describes whether points follow usually subjective patterns of regular, clustered, or random distribution. The NNI measures the distance between each point and its nearest neighbor’s location. All the nearest neighbor distances are averaged, and if the average distance is less than the average for a random distribution, the distribution of the features being analyzed is considered clustered. If the average distance is greater than a random distribution, the features are considered regularly dispersed. The index is expressed as the ratio of the mean observed distance divided by the expected distance, which is based on a random distribution with the same number of points covering the same total area,

1 $$\begin{aligned} NNI_i = \displaystyle \frac{\frac{1}{n} \sum _{j=1}^n d(i,j)}{0.5 \sqrt{HullArea(i)/n}}. \end{aligned}$$

Neurons in all layers except layer I and white matter tend more towards uniformly dispersed distribution, especially neurons of layer IV which tend more towards random distribution.

Depending on its position in the cortex, a neuron may be placed more toward the middle or more toward the edge of its layer. The computation of properties of its neighborhood may be confounded by reaching into adjacent layers and using neurons with different properties for the computation of statistics. To identify this case, measurements may be taken only from neurons found within the range of angle, or *slices*. Features measured in several directions can identify border neurons and changes in neuronal properties in different directions. Slices may be regarded as measurement units reaching from a single neuron, each unit representing a population of neighboring neurons found in a given direction from the central neuron. The relationship of different populations within an area has been extensively studied in the frame of biological diversity of species, landscapes and other^37,38^. Considering the neurons in a slice as members of a single *species*, and the *k* neighbors of a neuron as the population of all species in their habitat, biodiversity measures evaluate the relationship between the species. In this context, the number of slices is the number of different species, or *richness*, and the relative abundance of the different species in an area as *evenness*. The two most often used such measures are the Shannon index^39^ and Simpson index^40^. The Shannon index gives a quantitative measure of the uncertainty in predicting the species of an individual chosen randomly from the population. The Simpson index measures the probability that the two individuals who are randomly chosen (with replacement) from the total population will be of the same species.

2 $$\begin{aligned} Shannon = - \sum _{i=1}^{R} p_i \ln p_i = \ln \left( \frac{1}{\prod _{i=1}^{R} p_i^{p_i}} \right) , \quad Simpson = \sum _{i=1}^{R} p_i^2, \end{aligned}$$

where *R* is the number of different species or, here, slices, and $p_i$ is the proportion of species of the *i*th type in the population or proportion of neurons in *i*th slice to the number of the neurons in *k*-neighbourhood. If all slices have an equal number of neurons, $p_i$ values equal 1/*R*, and the Shannon index takes the maximum value of $\ln R$. If the numbers are unequal, the weighted geometric mean of the $p_i$ values is larger, which results in the index having smaller values. The index equals zero if the neurons from only one slice are present since there is no uncertainty in predicting the slice they are in. The index gives information about the relation between the number of types and the presence of the dominant type. The mean proportional abundance of the slices increases with decreasing number of slices and with the increasing abundance of the slice with the largest number of neurons, the index obtains small values in regions of high diversity like neurons on borders between the layers, thin layers, and especially layer I neurons. The index is large in homogeneous areas like the middle of layer III, where slices reaching from a neuron remain in the area of the layer.

### Experimental subjects statement

All specimens were collected during regular autopsies at pathology departments of the University of Zagreb, School of Medicine, approved by the Ethics Committee of the University of Zagreb, School of Medicine and in accordance with the Declaration of Helsinki, and informed consent was obtained from the next of kin.

## Results

**Figure 3:**
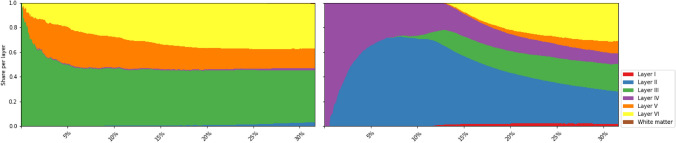
Neuron-centric analysis enables richer statistics. Shown here is the proportion of the top largest (left) and smallest (right) neurons found in the section, distributed per layer. The largest neurons were found in layer III of the cortex and were followed by neurons of layer V and layer VI. Out of the 50 largest neurons, $43 (86\%)$ were found in layer III, $5 (10\%)$ in layer V, and $2 (4\%)$ in layer VI. Out of the 500 largest neurons, $268 (54\%)$ were found in layer III, $142 (28\%)$ in layer V, $87 (17\%)$ in layer VI, and only $3 (1\%)$ in layer IV, which follows neuroanatomical observations.

The distribution of neurons’ features across the cortex provides insight into different aspects of the cytoarchitectonic organization. This detailed, neuron-level approach allows for tissue inspection following known cytoarchitectonic principles like, for instance, the distribution of the largest neurons. Those with the largest *area* were found in layer III of the cortex and were followed by neurons of layer V and layer VI. Out of the 50 largest neurons, $43 (86\%)$ were found in layer III, $5 (10\%)$ in layer V, and $2 (4\%)$ in layer VI. Out of the 500 largest neurons, $268 (54\%)$ were found in layer III, $142 (28\%)$ in layer V, $87 (17\%)$ in layer VI, and only $3 (1\%)$ in layer IV. This comparison confirms that the computed features yield meaningful results and follow neuroanatomical observations. Visualization of the ratio of the distribution of largest and smallest 500 neurons among the layers is shown in Fig. 3. Neuron *circularity* and *roundness* were found the lowest in layer VI which is known to consist of multipolar neurons with dendrites reaching in different directions. Variations in grayscale intensity were expressed differentially in the cortical layers. Neurons with the highest mean grayscale values were mostly found in layer I, showing low NeuN dye intake. Neurons with the lowest *median* were predominantly found in layer VI, in layer IV and the middle of layer III, sometimes referred to as layer IIIb. No conclusion was made or the reason found for neurons of layer VI having such large NeuN uptake properties that resulted in lower individual grayscale intensities. Measures regarding neuron shape such as area, circularity or perimeter were shown to provide more discriminative power, which is not unexpected since the findings in neuroanatomical research rely to large extent on the shape and size of neurons.

**Figure 4:**
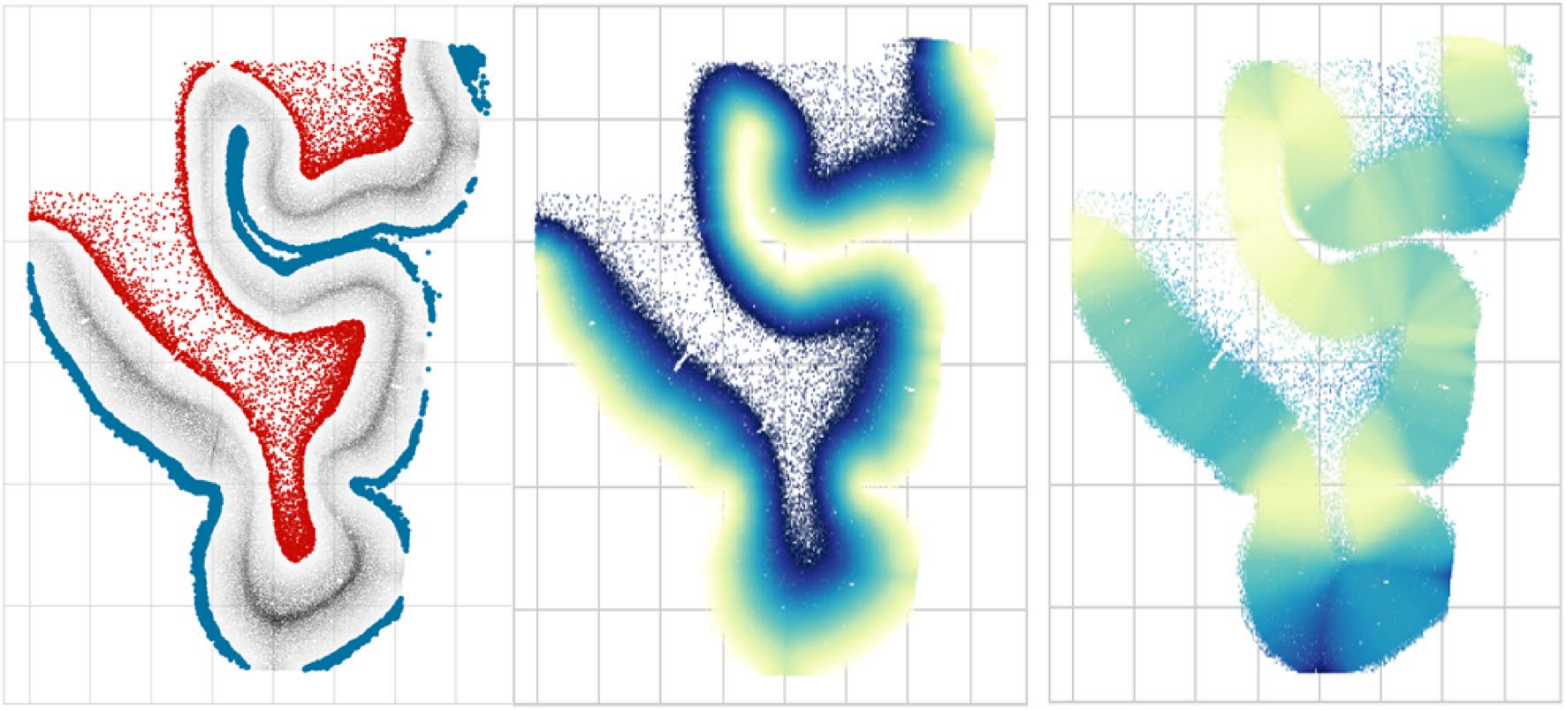
Features based on the local density and convex hull radius are used to obtain tissue features without drawing profiles and sampling perpendicular to the cortex. Left: sparse areas are separated into layer I and white matter. Middle: cortical depth. Right: cortical thickness.

Using local neuron density, layer I and white matter can be distinguished by having small neuron density, thus identifying *sparse regions* of the section, or *dense regions* containing layers II and IV, as shown in Fig. 2. The sparse region may further be split using the hull area feature—neurons in the white matter will have a large hull area, in contrast to the neurons of layer I, whose hull is bound between the border of the tissue and the dense layer II. By computing distances to layer I and white matter, cortical thickness and depth of each neuron are derived, as shown in Fig. 4.

### Machine learning pipeline

Although the developed neuron feature sets provide quantitative descriptors of cortical organization, they are not sufficient to provide a clear classification of the correct cortical layer. While some features may be more expressed in certain layers than in others, it is not straightforward to determine what exactly is changing between layers, or the impact and interconnection of different features. This led to an assumption that there is information contained in the developed features that can be analyzed, combined, and used to produce a precise classification of neurons concerning their location within the cortical layers through more complex and more expressive models. A supervised machine learning approach is used on a dataset of manually segmented layers in order to accurately predict the layer of each neuron in the histological section. Thus, layer segmentations can be obtained throughout the section. Feature attributions for the model are investigated to identify informative tissue features.

**Figure 5:**
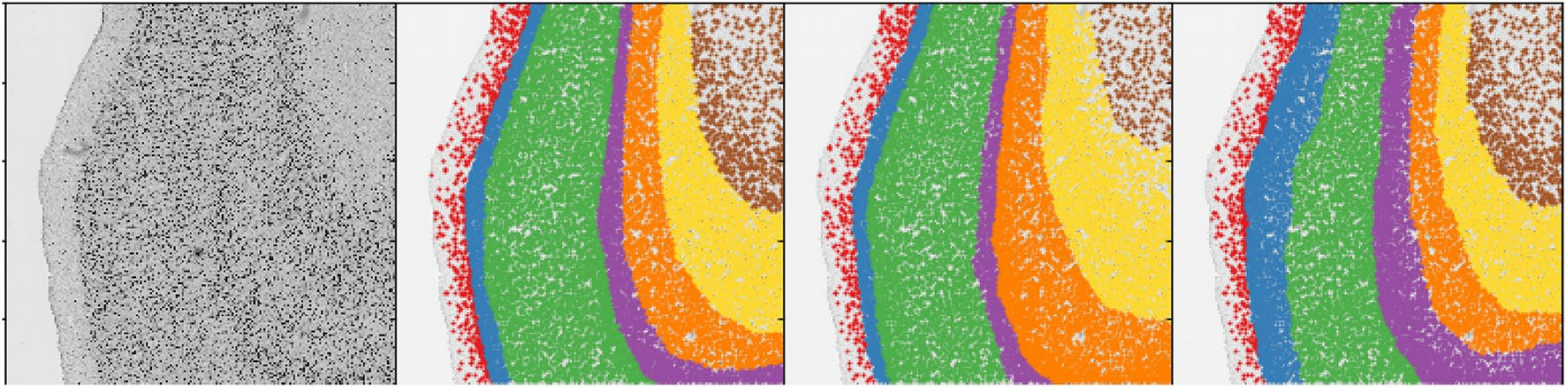
A detail of cortical layers manually delineated by the experts. Significant disagreement is observed on the boundaries of the layers, as well as on positioning the boundary between the cortex and the white matter.

To obtain the training dataset from which the machine learning method will learn to classify neurons according to their layers, portions of both digitized histological sections were given to three human experts in histology and cytoarchitectonics who manually delineated borders between the layers of the cortex. The apparent inconsistencies and mutual disagreement between the experts, as seen in Fig. 5, show the presence of experts’ bias. The experts disagreed on the boundaries of all layers, except on the very apparent layer I/layer II boundary. The manually labeled dataset contained 12,647 neurons in the $10\;\upmu \hbox {m}$ section and 9821 neurons in the $10\;\upmu \hbox {m}$ section.

**Figure 6:**
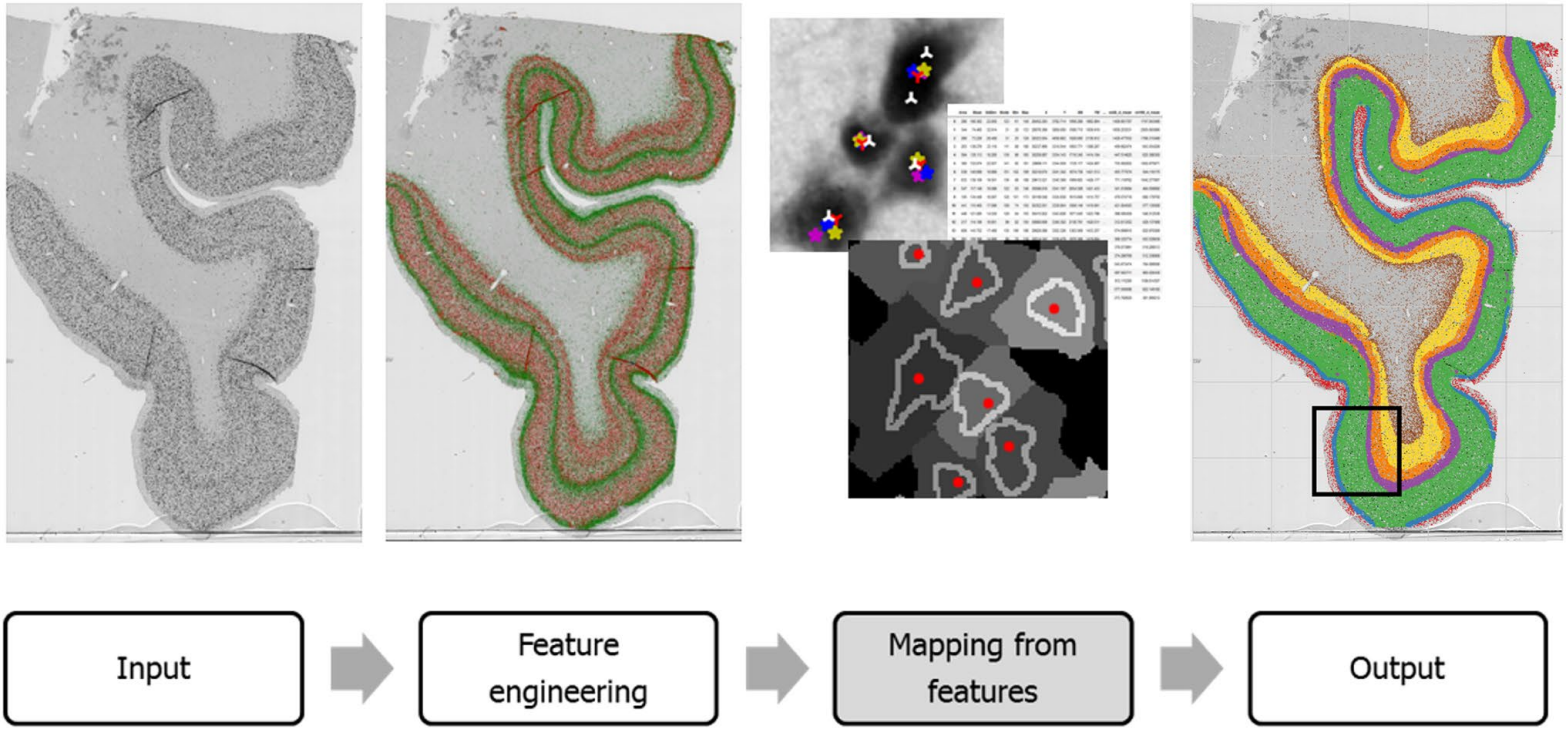
Learning to map neuronal phenotype to cortical layers using a classical machine learning pipeline. The neuron representation, or phenotyping, is based on automatic neuron segmentation and analysis of morphological and textural features. This representation is then used as an input to a machine learning model which learned to solve the multiclass classification task by classifying neurons among the six layers of the cortex. The model learned the variations in neuronal features and was able to generalize, i.e. create consistent and sensible predictions of layers across the whole histological section. The black rectangle frames the portion of the section that was manually labeled by three experts and used as training data.

Boosted decision trees, a state-of-the-art supervised learning method on tabular input data such as the computed neuron features^41,42^ were chosen for prediction and interpretation of cortical lamination for its several advantages. Decision trees mirror human decision-making more closely than other approaches^43^, which is especially useful when modeling human activities, such as the manual delineation of cortical layers, a decision-making process based on a combination of information about neurons’ characteristics. We used CatBoost^44^, a method based on gradient boosting over decision trees which is one of the most successful models for dealing with tabular data. The model was trained for 100 iterations with a learning rate of 0.1 and default other parameters. The best generalizations were obtained by combining the manual labels of all three raters in an ensemble. Three separate models were trained, one for each rater, and using softmax objective output probabilities were summed, and the final prediction was made using a maximum over all classes for each rater. Results of this approach are shown in the right of Fig. 6. Classes of neurons are predicted, and neurons are accurately classified in a way that follows the laminar pattern of the cortex.

Experiments with different sets of features have shown that although some combinations of features do achieve high accuracy on training data, that itself does not guarantee that the model will perform well on the whole histological section. The introduction of features based on the distance to sparse or dense regions has significantly improved the model’s ability to separate regions of the sections into parcels following the laminar layout of the cortex.

### Performance analysis

Without the existence of single ground truth for reference, the measurement of the model’s performance is considered in the context of inter-rater variability. Training data was split into $75\%$ training and $25\%$ test subsets, and predictions of the model were compared with the experts’ manual labels. Comparing neuron layer predictions, average agreement between two experts was $0.755 \pm 0.049$ for $10\;\upmu \hbox {m}$ and $0.809 \pm 0.049$ for $20\;\upmu \hbox {m}$ histological section. The average accuracy of the model, when compared to the three experts, was $0.872\pm 0.042$ and $0.897 \pm 0.047$. One could relate this to the accuracy in Wagstyl’s segmentation approach on the BigBrain, where the cross-validation, average per-point accuracy on the test fold was $0.83 \pm 0.02$.

## Discussion

### Analysis of individual feature attribution

In this study, we proposed a novel approach to analyzing cortical features in order to facilitate more detailed and specific scientific investigations. Currently, a gold standard is a manual annotation by trained experts. However, human experts are often biased, and results obtained in the such analysis are often inconsistent. An important feature in delineating cortical layers and areas is neuronal size. Our findings demonstrated that in analyzed regions, which belong to the homotypic isocortex of the prefrontal cortex, neurons in layer III are in general larger than neurons in layer V. Although a general sense is that neurons in the layer V are larger than neurons in layer III that is not true for the prefrontal cortex. The largest pyramidal neurons in the cerebral cortex are indeed found in layer V (Betz’s cells of the motor cortex), however, in most cortical areas pyramidal neurons in layer V are smaller than neurons in layer III^3^. This finding confirms that the computed features yield meaningful results and follow neuroanatomical observations.

**Figure 7:**
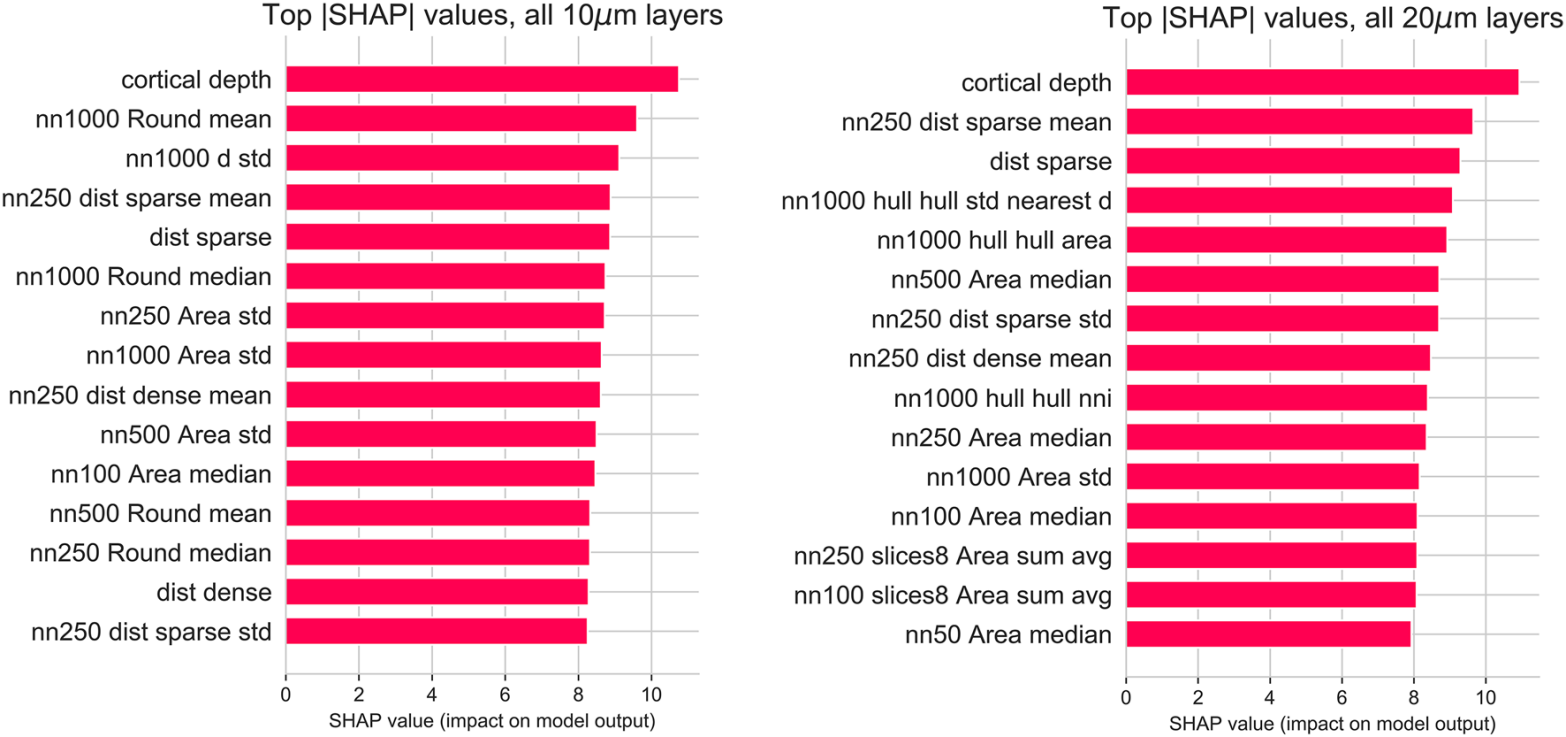
Importance of top 15 neuron features at the model level using the SHAP feature importance analysis for $10\;\upmu \hbox {m}$ (left) and $20\;\upmu \hbox {m}$ (right) sections. For the features that use properties of neighboring neurons, the number of the nearest neighbors is indicated at the start of the feature name. Regardless of differences in neuronal density, models on both sections preferred approximately a similar number of neurons in the nearest neighborhoods which were on average 500 neurons. For both sections, the cortical depth of a neuron was the most informative feature for machine learning models. Models in both sections also relied on features related to the size (Area) of neighboring neurons, as well as distances to areas of uniformly lower and higher densities. Individual shape and texture properties of neurons that do not take into account the neurons’ neighborhoods were the least informative and added no predictive value.

For a deeper understanding of both the model and features being used in the pipeline, an investigation of the impact of features on the prediction of the neuron’s class was performed on both global (model) and instance (individual neuron) levels. A recent approach for measuring feature attributions in learning models, the SHAP measure^45^, was used for the estimation of neuron features that contribute the most to neuron classification within the layers. Details on SHAP values and their influence on model outputs for both $10\;\upmu \hbox {m}$ and $20\;\upmu \hbox {m}$ datasets are presented in Fig. 7, and in detail for each cortical layer in both sections in the Supplementary Information file, Fig. S.1 and Fig. S.2.

**Figure 8:**
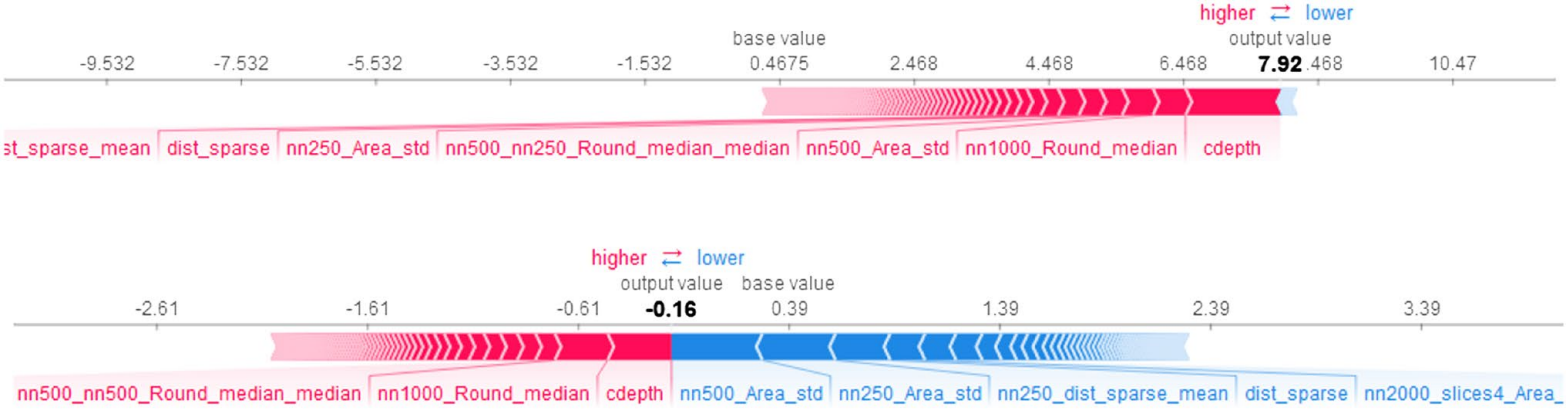
Contribution of different features for predicting a neuron’s layer can be analyzed at the instance-level, for each individual neuron. Top: neuron features of a single neuron of layer III that contributed to the increase from the base SHAP value and making the prediction. Bottom: the importance of features that decreased the output value for prediction of the same neuron as a layer II neuron.

An important aspect of this approach is the ability to identify features that contribute to predicting a single instance of the data, for each neuron. For instance, Fig. 8 shows which neuron features for a neuron of layer VI contributed to the increase of the base SHAP value and making the prediction. The figure also shows the impact of features that decreased the output value for the prediction of the same neuron as a white matter neuron.

The cortical depth feature had a large impact on the model’s output. This is because it helps integrate simpler features like local densities with anatomical observations regarding the position of a neuron within the cortex. Features based on oriented measurements that measure the change of cytoarchitectonic properties in different directions also had considerable feature importance, being able to identify neurons on the border of cortical layers. It was shown that building on lower-level features yields features that have greater discriminative power and thus greater importance. This is due to their capacity to overcome local variations in neuron features and take, for instance, the mean of those features. In contrast, using features of a neuron such as an area, there is no increase in the accuracy of prediction, which is reflected in the low importance of these features. This is probably why different methods for local pattern analysis and classical image feature extraction methods are not very successful in cortical layer segmentation. Range of variability radius was established, giving an estimate of the size of the neuron’s neighborhood in which measurements should be made, so it is large enough to overcome local variations in neuron distribution and recognize its location within the cortical structure on one hand, and on the other narrow enough so that measurements are not confounded by reaching too far into adjacent layers.

To investigate the effects of section thickness, we have analyzed the number of nearest neighbors in several fixed ranges and established that in $10\;\upmu \hbox {m}$ section $55.6\%\pm 0.7\%$ fewer neurons are found, compared to the $20\;\upmu \hbox {m}$ sections. On the other hand, one can observe the number of nearest neighbors in the top most informative features selected by the models. Observing the top 20 features in models trained on each section, an increase in the average number of neighbors in features can be noted in the $10\;\upmu \hbox {m}$ section (630 compared to 580), however, the difference is not statistically significant. Both results support the reasoning in the Materials and Methods section about the hypothetical range in which nearest-neighbor values should be computed, and that one should prefer the number of neighbors over predefined ranges.

During experiments with different models, it was noticed that parts of layer III and layer VI are sometimes divided into sub-layers that follow the direction of the laminar structure, although being very short and not extending through a significant portion of the slice. Further investigations using the developed methodology may provide more detailed insight into the sub-layering of cortical structure.

### Limitations

The proposed method has demonstrated the ability to generalize across sections with a very limited training dataset, showing promising results and indicating that it could be transferable to other brains. However, due to the complexity of the brain, further research using larger amounts of histological data of greater variability is needed to demonstrate with certainty the degree to which these results can be generalized across different brains. This will also allow for further testing of the approach in different brain areas, tissue staining, and cutting planes. In oblique slicing, where the angle of the slicing plane may influence the shape of the neurons, for instance, the pyramidal neurons will not appear as triangles if the slicing was perpendicular to the neuronal columns. This limitation may be overcome by using the 3D representation of neurons.

### Conclusion

We have proposed a new methodology for modeling brain cytoarchitectonics which builds from the cellular level and infers about larger structures by creating data-driven, neuron-level tissue descriptors based on features of individual neurons, or neuronal phenotyping. This is in contrast to today’s other approaches in neuroscience, which are mostly based on pixel data. The movement from pixel-wise towards neuron-centric analysis, in which the structure of the brain is studied through the lens of the relationship between the neurons, in contrast to relying solely on changing values of pixels in the histological image, conveys a new paradigm in the field and enables methods from other disciplines to be introduced. Here, we refer to the shift in the way histological data is gathered, examined, and comprehended, and the introduction of machine learning methods that operate on tabular data, for which the neuron representations had to be made first. By leaning more on data-driven methods, our approach lowers the need for human-dependent interventions and interpretations, which allows for more objective and reproducible quantification on a large scale. These settings enable novel insights into the organization of cortical microstructure and subtle differences in neuropathology. By enabling the emergence of new, better descriptions and understanding of the brain structure in different areas and stages of development, our work facilitates movement towards fully automatic, high-throughput, objective investigations, allowing the processing of ever-larger amounts of histological data available globally in research centers today.

The scenario of using the proposed methodology was demonstrated on a particular brain region and validated using a set of data manually labeled by three experts. Our methodology is easily extensible with novel neuronal features such as different stainings or receptor maps and allows the use of other machine learning-based computational methods, such as graph neural networks, which will entice future research initiatives in the field of computational neuroscience.

## Supplementary Information

Supplementary Figures.

## Data Availability

The datasets generated during and/or analyzed during the current study are available from the corresponding author upon reasonable request.
