# Supplementary material for: Interpretable machine learning approach for neuron-centric analysis of human cortical cytoarchitecture
Source: Sci Rep. 2023 Apr 5;13:5567. doi: 10.1038/s41598-023-32154-x (PMC10076420; doi:10.1038/s41598-023-32154-x)
Supplement: Supplementary file 1 — Supplementary Figures. [file 41598_2023_32154_MOESM1_ESM.pdf]

Interpretable Machine Learning Approach for Neuron-Centric Analysis of Human Cortical Cytoarchitecture

Supplementary Information

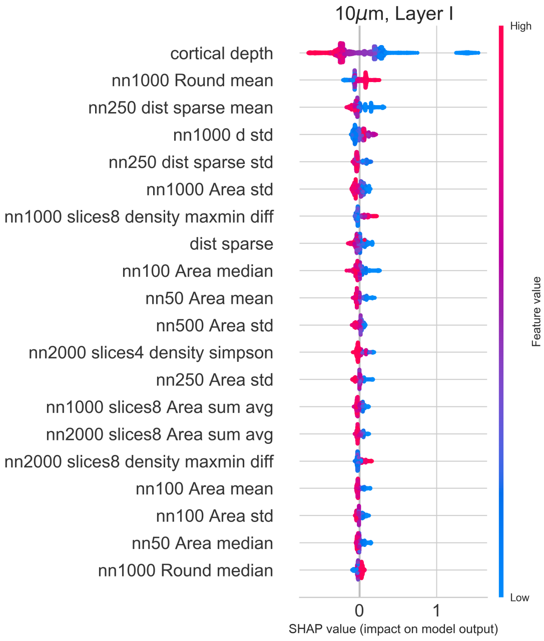

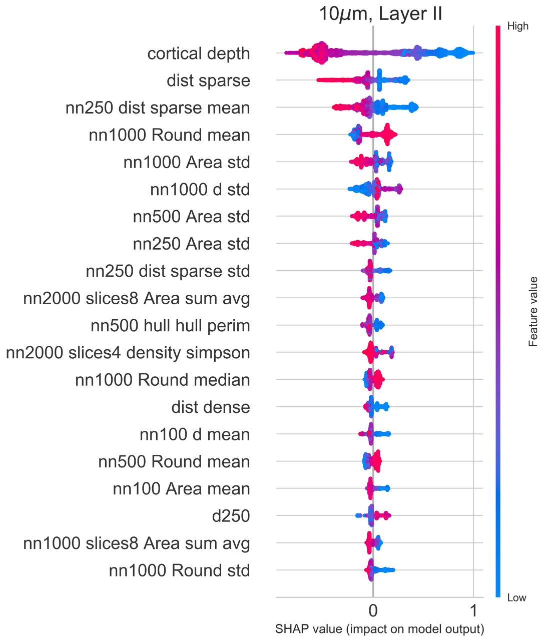

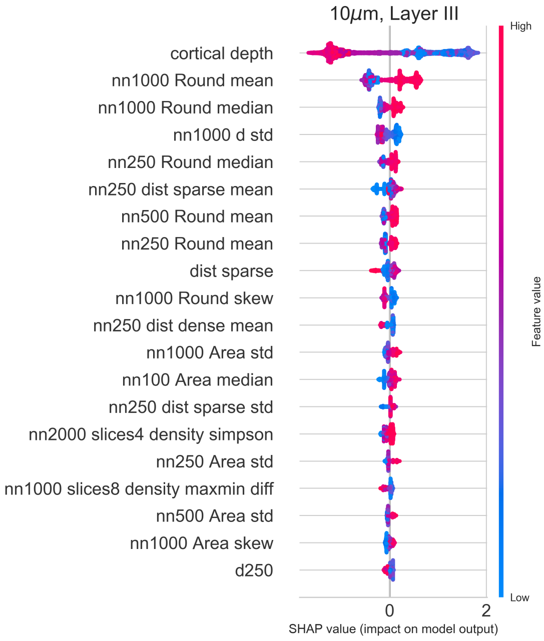

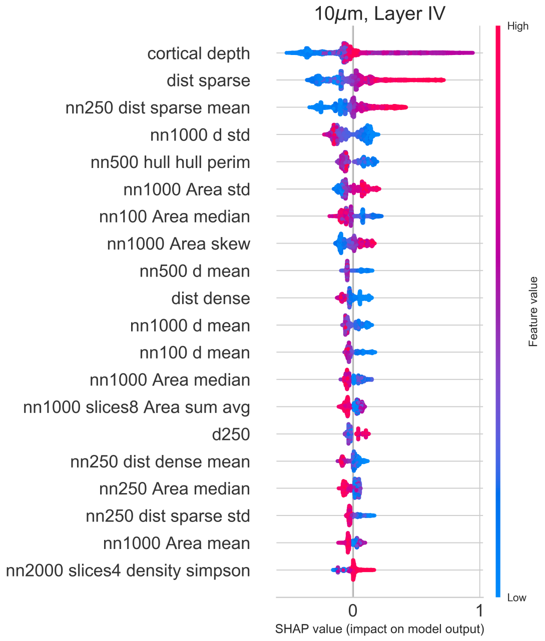

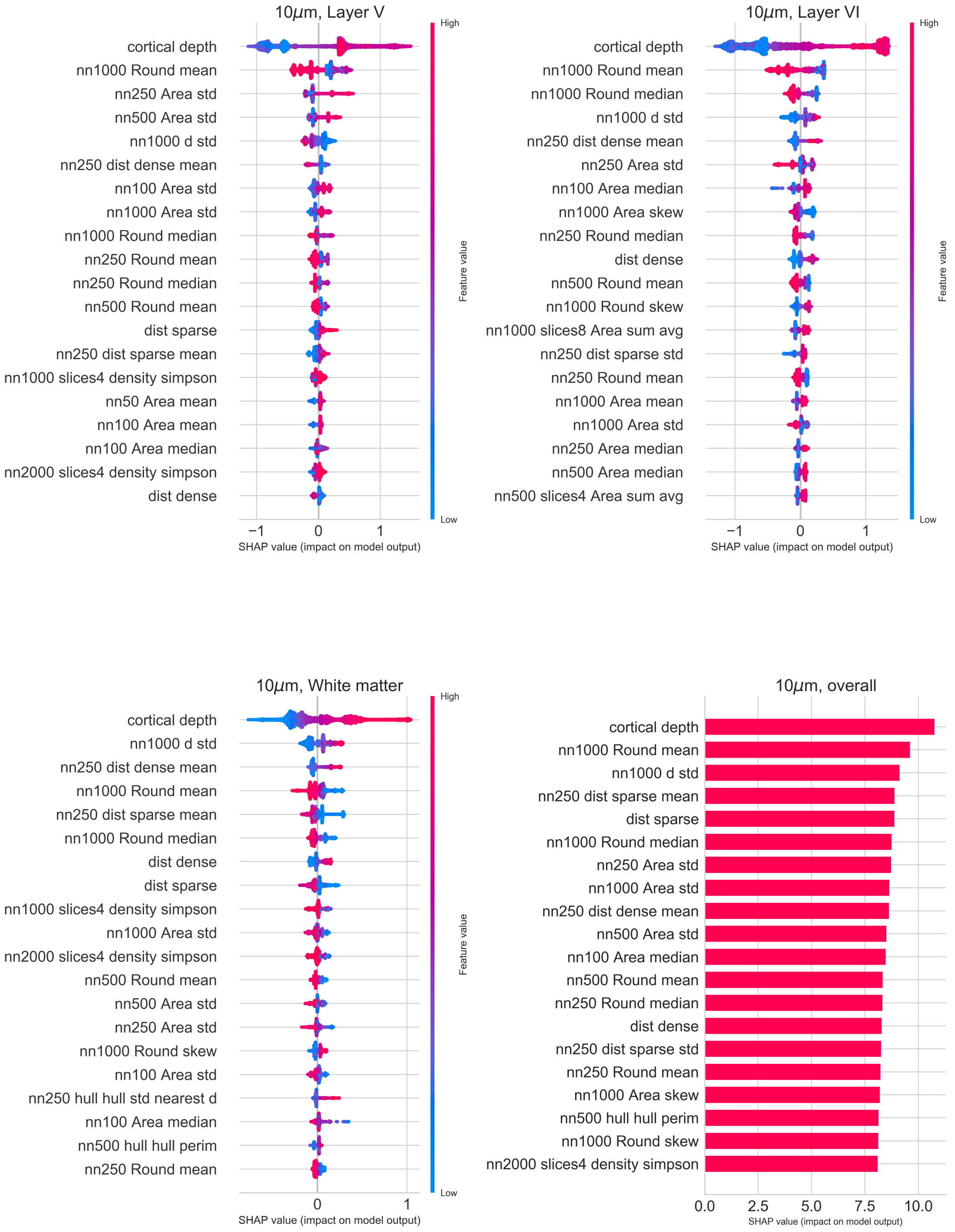

Figure S.1: The importance of neuronal phenotype features in classification models, measured by SHAP feature importance for top 20 features for each layer in the 10μm section. At the start of each feature name, it is indicated whether the feature is using information from the neuron's neighborhood and the size of the neighborhood. At the end of the feature, name is usually a statistic that was computed using the measure indicated in the middle of the feature name. For example, `'nn500 Area std'` indicates the standard deviation of Areas in neighboring 500 neighbors. Higher SHAP values indicate that the particular feature has a higher impact on the prediction output, meaning that the feature has a greater impact on the model's prediction.

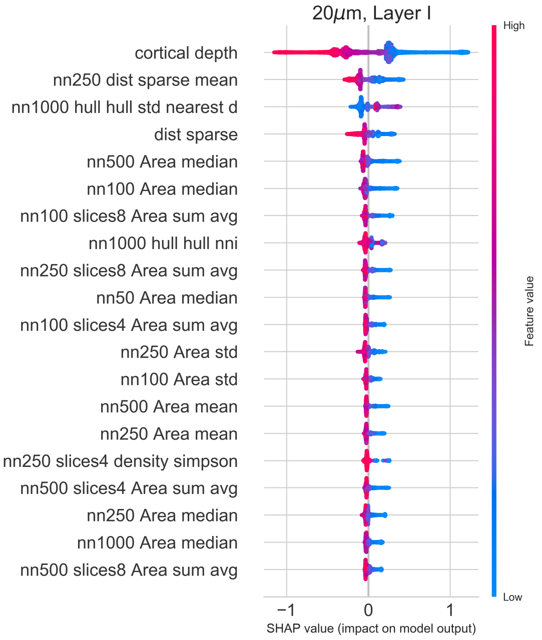
20μm, Layer I
cortical depth
nn250 dist sparse mean
nn1000 hull hull std nearest d
dist sparse
nn500 Area median
nn100 Area median
nn100 slices8 Area sum avg
nn1000 hull hull nni
nn250 slices8 Area sum avg
nn50 Area median
nn100 slices4 Area sum avg
nn250 Area std
nn100 Area std
nn500 Area mean
nn250 Area mean
nn250 slices4 density simpson
nn500 slices4 Area sum avg
nn250 Area median
nn1000 Area median
nn500 slices8 Area sum avg
−1
0
1
SHAP value (impact on model output)
High
Low
Feature value

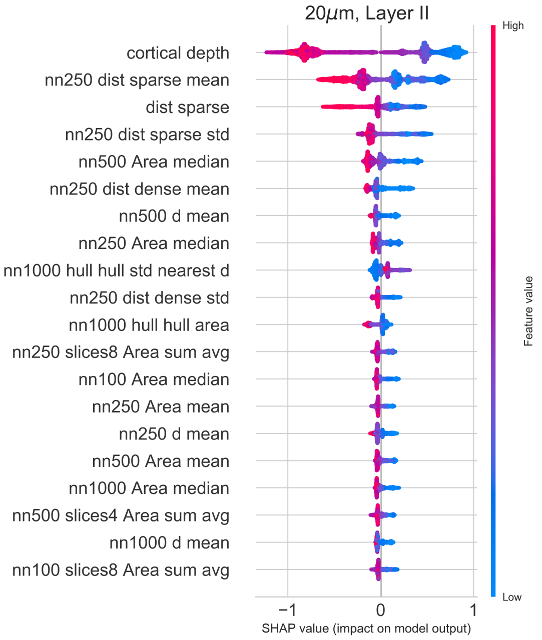
20μm, Layer II
cortical depth
nn250 dist sparse mean
dist sparse
nn250 dist sparse std
nn500 Area median
nn250 dist dense mean
nn500 d mean
nn250 Area median
nn1000 hull hull std nearest d
nn250 dist dense std
nn1000 hull hull area
nn250 slices8 Area sum avg
nn100 Area median
nn250 Area mean
nn250 d mean
nn500 Area mean
nn1000 Area median
nn500 slices4 Area sum avg
nn1000 d mean
nn100 slices8 Area sum avg
−1
0
1
SHAP value (impact on model output)
High
Low
Feature value

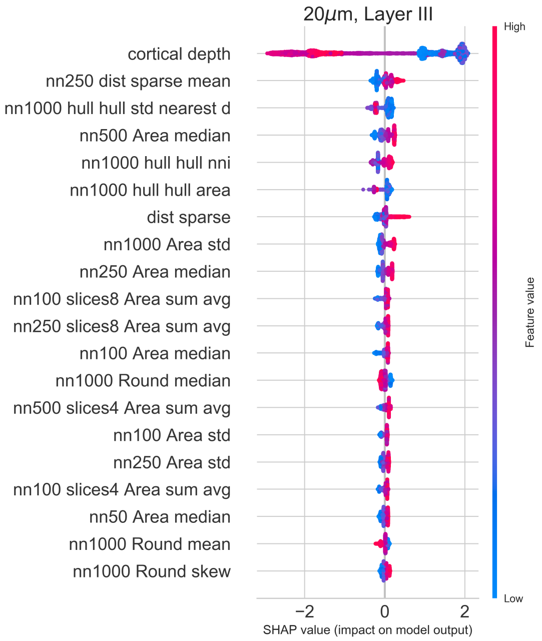
20μm, Layer III
cortical depth
nn250 dist sparse mean
nn1000 hull hull std nearest d
nn500 Area median
nn1000 hull hull nni
nn1000 hull hull area
dist sparse
nn1000 Area std
nn250 Area median
nn100 slices8 Area sum avg
nn250 slices8 Area sum avg
nn100 Area median
nn1000 Round median
nn500 slices4 Area sum avg
nn100 Area std
nn250 Area std
nn100 slices4 Area sum avg
nn50 Area median
nn1000 Round mean
nn1000 Round skew
−2
0
2
SHAP value (impact on model output)
High
Low
Feature value

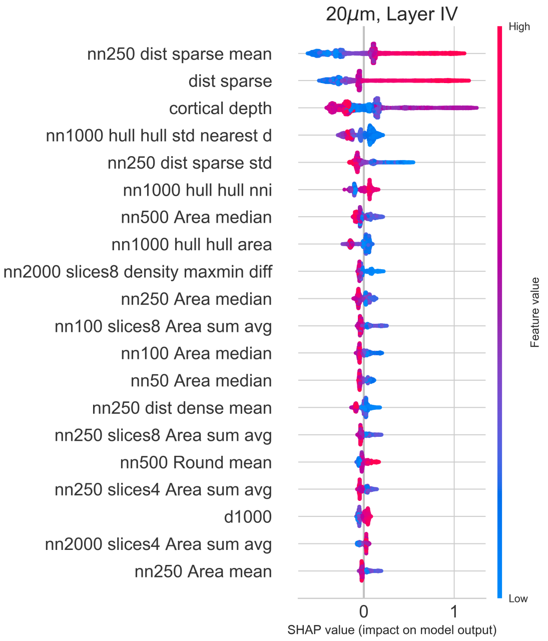
20μm, Layer IV
nn250 dist sparse mean
dist sparse
cortical depth
nn1000 hull hull std nearest d
nn250 dist sparse std
nn1000 hull hull nni
nn500 Area median
nn1000 hull hull area
nn2000 slices8 density maxmin diff
nn250 Area median
nn100 slices8 Area sum avg
nn100 Area median
nn50 Area median
nn250 dist dense mean
nn250 slices8 Area sum avg
nn500 Round mean
nn250 slices4 Area sum avg
d1000
nn2000 slices4 Area sum avg
nn250 Area mean
0
1
SHAP value (impact on model output)
High
Low
Feature value

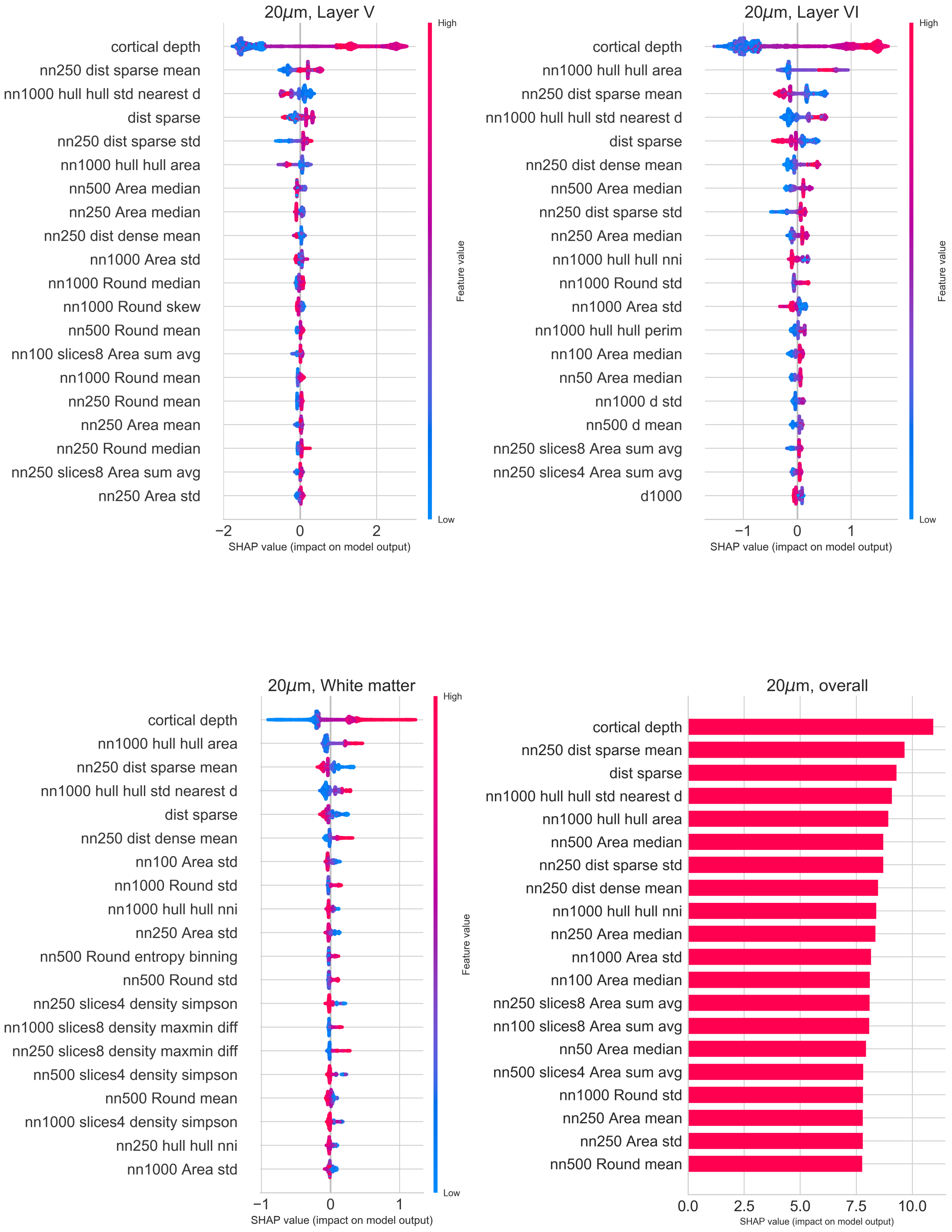

Figure S.2: The importance of neuronal phenotype features in classification models, measured by SHAP feature importance for top 20 features for each layer in the 20μm section. At the start of each feature name, it is indicated whether the feature is using information from the neuron's neighborhood and the size of the neighborhood. At the end of the feature, name is usually a statistic that was computed using the measure indicated in the middle of the feature name. For example, `'nn1000 hull hull area'` indicates the hull area of the convex hull of neighboring 1000 neighbors. Higher SHAP values indicate that the particular feature has a higher impact on the prediction output, meaning that the feature has a greater impact on the model's prediction.
